# An overview report on the application of heteropoly acids on supporting materials in the photocatalytic degradation of organic pollutants from aqueous solutions

**DOI:** 10.7717/peerj.5501

**Published:** 2018-09-12

**Authors:** Ali Nikoonahad, Babak Djahed, Samira Norzaee, Hadi Eslami, Zahra Derakhshan, Mohammad Miri, Yadolah Fakhri, Edris Hoseinzadeh, Seyed Mehdi Ghasemi, Davoud Balarak, Reza Ali Fallahzadeh, Mansur Zarrabi, Mahmoud Taghavi

**Affiliations:** 1 Department of Environmental Health Engineering, School of Health, Ilam University of Medical Sciences, Ilam, Iran; 2 Department of Environmental Health Engineering, Iranshahr University of Medical Sciences, Iranshahr, Iran; 3 Department of Environmental Health Engineering, Rafsanjan University of Medical Sciences, Rafsanjan, Iran; 4 Department of Environmental Health Engineering, Larestan University of Medical Sciences, Larestan, Iran; 5 Cellular and Molecular Research Center, Department of Environmental Health Engineering, School of Public Health, Sabzevar University of Medical Sciences, Sabzevar, Iran; 6 Student Research Committee, Department of Environmental Health Engineering, School of Public Health, Shahid Beheshti University of Medical Sciences, Tehran, Iran; 7 Department of Environmental Health Engineering, Tarbiat Modares University, Tehran, Iran; 8 Deputy of Health, Babol University of Medical Sciences, Babol, Iran; 9 Department of Environmental Health Engineering, Zahedan University of Medical Sciences, Zahedan, Iran; 10 Department of Environmental Health Engineering, Shahid Sadoughi University of Medical Sciences, Yazd, Iran; 11 Department of Environmental Health Engineering, Alborz University of Medical Sciences, Karaj, Iran; 12 Department of Environmental Health Engineering, School of Public Health, Social Development & Health Promotion Research Center, Gonabad University of Medical Sciences, Gonabad, Iran

**Keywords:** Heteropolyanions, Phosphotungstic acid, Photocatalytic activity, Polyoxometalate

## Abstract

Organic pollutants contaminate water resources and the environment when discharged into water streams. Also, the presence of these materials in incompletely treated or untreated wastewater leads to serious environmental hazards. The hydroxyl radicals and holes are regarded as the most oxidant species in the degradation of organic pollutants using the studied composites. The results of this review show that heteropoly acids on supporting materials could be considered as appropriate photocatalysts in the removal of organic pollutant from aqueous solutions.

## Introduction

Organic pollutants contaminate water resources and the environment when discharged into water streams. Also, the presence of these materials in incompletely treated or untreated wastewater leads to serious environmental hazards. Therefore, developing some technologies that can destruct these pollutants is of great importance. Several common processes such as condensation, ultrafiltration, membrane separation, and adsorption are applied for removing the organic pollutants. However, these methods cannot completely degrade the pollutants into nontoxic substances; rather, they often are just responsible for transferring these substances to other phases (Yang et al., 2012). In this regard, photocatalytic degradation can be regarded as one of the promising technologies to solve this problem (Lu et al., 2013; Norzaee et al., 2017). During the last decade, heterogeneous photocatalysis has received much attention as an unconventional technology in environmental remediation because of its advantages such as applying mild experimental conditions; i.e., atmospheric pressure and room temperature (Marcì, García-López & Palmisano, 2014). This technique can degrade most of the organic pollutants and mineralize them to final products such as carbon dioxide, water, and other small inorganic molecules (Feng, Shang & Liu, 2014).

In recent years, much research has been conducted into improving the photocatalytic process by preventing/delaying the recombination of the hole-electron pair at the surface of the photocatalyst, enhancing the electron transfer rate (Li, Vorontsov & Jing, 2015; Taghavi et al., 2018a), increasing the specific surface area and porosity of the photocatalyst by introducing a structure directing agent (Feng, Shang & Liu, 2014), enhancing the amount of adsorbed photons by photocatalyst at UVA and visible region, and reducing energy consumption through using LEDs or sunlight as the light source. In addition, some attempts have been made to use sensitizers on the surface of photocatalyst or couple with other semiconductors in order to modify the physiochemical and electronic properties of photocatalyst (Marcì, García-López & Palmisano, 2014). Heteropoly acids (HPA), as green and eco-friendly catalysts, have been proposed as potential candidates to be used as surface modifiers of TiO_2_ intended for photocatalytic degradation (Hakimi et al., 2014; Li, Vorontsov & Jing, 2015). The compound catalyst could eliminate the difficulties for recycling heteropoly acids and prevent the recombination of hole-electron pairs for both catalysts when heteropoly acids were carried on semiconductors such as TiO_2_. Thus, an increase in the specific area of heteropoly acid and synergy resulted in enhancing the photocatalytic degradation (Wang, Huang & Yang, 2010). Among heteropoly acids, Keggin-type heteropoly acids have been widely implemented for supporting materials such as semiconductors, resins, and clays and for environmental remediation (Guo & Hu, 2007; Leal Marchena et al., 2015; Lei et al., 2005; Shi et al., 2012; Wei et al., 2012; Zhang et al., 2015). The chemical formula of these acids is [XM_12_O_40_]^n−^, where X indicates the heteroatom such as P^5+^ or Si^4+^ and M displays the addenda atom, mostly W or Mo in high oxidation state (Tabatabaee et al., 2015).

The present study was conducted to review the application of Keggin-type heteropoly acids on supporting materials in the photocatalytic degradation of organic pollutants in aqueous solutions.

## Survey Methodology

Available reports on the photocatalytic application of Keggin-type heteropoly acid-based composites were searched. For this purpose, important international and national databases were considered for retrieving all related studies; hence, Google Scholar, Science direct, Scopus and PubMed databases were considered to survey peer-reviewed journal articles. The search terms were selected as “Keggin-type heteropoly acid”, “ *α*–Keggin anions”, “Keggin-type polyoxometalate”, “phosphotagustic acid”, “phosphomolybdic acid”, together with “photocatalytic activity,” “photocatalytic degradation,” “degradation” and “removal”. We strictly searched for publications focusing on the applications of Keggin-type heteropoly acid–based composites in photocatalytic degradation. Research articles that were published before March 2017 were collected based on our search criteria.

## Results

### Photocatalytic activity

The photocatalytic degradation activity of synthesized nanophotocatalysts was compared with that of other photocatalysts reported in the literature for photocatalytic degradation of various pollutants (Table 1). Based on the obtained results, the synthesized nanophotocatalysts could represent a reasonable photocatalytic degradation activity of organic pollutants, compared to other photocatalysts.

**Table 1:** Photocatalytic activity of Keggin-type heteropoly acids on supporting materials in photocatalytic degradation of organic pollutants in aqueous solutions.

Catalyst	Pollutant	Light source	V(mL)	pH	Dose	Initial concentration	Time (min)	R(%)	Specific condition	Ref
H_3_PW_12_O_40_/TiO_2_	Congo Red	A 400 W Xe lamp (*λ* >420 nm)	200 mL	–	250 mg	50 mg/L	120	92	–	Yang et al. (2005)
	Methyl Orang						240	72.4		
	Ponceau G						180	94.8		
	Orange II						240	67.2		
	Eriochrome Blue Black B						180	75.8		
	Alizarin S						240	72.8		
	Methylene Blue						60	96		
	Neutral Red						60	98.2		
	Rhodamine B						60	98		
	Fuchsin Acid						240	75		
H_3_PMo_12_O_40_/TiO_2_	Methylene Blue	UV-A (*λ*_max_ = 365 nm)	–	–	–	–	30	90	–	Nivea et al. (2014)
H_3_PW_12_O_40_/TiO_2_								62		
Ce- H_3_PMo_12_O_40_/TiO_2_	Methylene Blue	A 125 W high-pressure Hg lamp	50 mL	–	200 mg	40 mg/L	100	98	–	Shi et al. (2012)
La- H_3_PW_12_O_40_/TiO_2_								96		
H_3_PW_12_O_40_/TiO_2_	Parathion-methyl	Visible light	120 mL	–	200 mg	50 mg/L	40	>95	–	Guo & Hu (2007)
PW_11_–SiO_2_ film	Rhodamine B	A 125 W high-pressure Hg Lamp (*λ*_max_ = 313.2 nm)	150 mL	–	1. 25 ×12 × 45 mm	1 mM/L	240	70	–	Yang et al. (2003b)
	Erythrosine BS						90	98.6		
	Methyl Orange						240	42.8		
	Congo Red						240	59.4		
PW_11_–TiO_2_ film	Rhodamine B						240	87		
	Erythrosine BS						90	99.4		
	Methyl Orange						240	55.6		
	Congo Red						240	73.2		
HPW-yttrium-TiO_2_	Methyl Orange	A 300 W Xe lamp (*λ*>365 nm)	50 mL	1	30 mg	10 mg/L	21	100	–	Yajun, Kecheng & Changgen (2011)
Immobilized H_3_PW_12_O_40_ (30%) on NH_4_ZSM5 zeolite	Methyl Orange	A 125 W high-pressure Hg lamp (*λ*_max_ = 365 nm)	400 mL	2.5	0.75 g/L	2.62 mg/L	240	91	–	Leal Marchena et al. (2015)
H_3_PW_12_O_40_/TiO_2_	Congo red	A 150 W Xe arc lamp	10 mL	–	12 mg	10 μ M	30	32	–	Pearson, Bhargava & Bansal (2011a)
H_3_PW_12_O_40_/TiO_2_/Cu								58		
H_3_PW_12_O_40_/TiO_2_/Ag								71		
H_3_PW_12_O_40_/TiO_2_/Pt								79		
H_3_PW_12_O_40_/TiO_2_/Au								86		
H_3_PW_12_O_40_/MCM-41	Imidacloprid	A 300 W Xe light (equipped with 365 nm optical filter, *λ*_max_ = 365 ±10 nm)	50 mL	–	20 mg	10 mg/L	300	58	–	Feng, Li & Liu (2012)
H_3_PW_12_O_40_/BiVO_4_	Methylene blue	A 500 W Xe lamp (with UV cut-off filters, *λ*>420 nm)	50 mL	–	30 mg	10 mg/L	360	93	–	Zhang et al. (2013)
H_3_PW_12_O_40_/TiO_2_	Methyl orange	A 300 W medium-pressure Hg lamp (*λ*_max_ = 365 nm)	20 mL	2	10 pieces glass slide (12.7 × 38.1 mm^2^/piece)	5 mg/L	60	93.4	–	Niu & Hao (2011)
H_3_PW_12_O_40_/TiO_2_/Float pearls	Congo red	A 250 W medium-pressure Hg	100 mL	7	1.5 g/L	60 mg/L	70	90	With aeration 2L/min	Yang et al. (2008)
H_3_PW_12_O_40_ pillared Mg_3_Al-LDH	Methyl orange	UV light	150 mL	–	60 mg	0.02 M	30	96.39	With H_2_O_2_	Zhang et al. (2011)
Zeolite-Y/ TiO_2_/Co^2+^/H_3_PMo_12_O_40_	Methyl orange	Two 200 W tungsten filament lamps	10 mL	–	75 mg	5 mg/L	240	51	In presence of ethanol	Dubey et al. (2006)
H_3_PW_12_O_40_/TiO_2_ film	Rhodamine B	A 300 W Xe lamp (with an IR cut filter, *λ* = 320–780, 200 mW/cm^2^)	120 mL	4.4	two pieces of quartz (4.5) mg	25 mg/L	240	>98	–	Lu et al. (2012)
H_3_PW_12_O_40_/TiO_2_	p-Nitroaniline	Two 125 W medium-pressure Hg lamps (*λ*_max_ = 365 nm)	100 mL	3	0.6 g/L	10 mg/L	120	95.11	–	Huang & Liu (2011)
H_3_PW_12_O_40_/Polymethylmethacrylate/ Polycaprolactam nanofibrous membrane	Methyl orange	A 300 W high-pressure Hg lamp	50 mL	1	–	10 mg/L	30	92.7	–	Li et al. (2016)
H_3_PW_12_O_40_/TiO_2_/SiO_2_	Methyl violet	A 500 W Xe lamp (intensity: 1,200 µmol/m^2^.s)	–	3	2.9 g/L	10 mg/L	150	95.4	–	Yang et al. (2016)
	Methyl orange							99.9		
	Methyl red							100		
	Naphthol green B							93.7		
	Methylene blue							81		
H_3_PW_12_O_40_/ZrO_2_	4-nitrophenol	A 50 W high-pressure Hg lamp	100 mL	–	100 mg	0.36 mM/L	90	>90	–	Qiu, Zheng & Haralampides (2007)
	Methylene blue					0.065 mM/L		>90		
H_3_PMo_12_O_40_/MnO_2_	Methylene blue	UV light (*λ*_max_ = 365 nm)	100 mL	4	50 mg/L	32 mg/L	150	>98	–	Kannan et al. (2011)
H_3_PW_12_O_40_/ZrO_2_	Methylene blue	A 400 W high-pressure Hg lamp	10 mL	1.16	20 mg	10 mg/L	15	87	Oxygen flow rate of 5 mL/min	Salavati, Tavakkoli & Hosseinpoor (2012)
	Congo red			6.15		20 mg/L		84		
	Rhodamin B			1.27		30 mg/L		87		
	Bromothymol Blue			1.1		20 mg/L		52		
	Alizarin			6.6		40 mg/L		61		
H_3_PW_12_O_40_/Ag-TiO_2_	Atrazine	A 300 W Xe lamp (equipped with an IR cut filter, intensity: 200 mW/cm^2^)	100 mL	3.4	1 g/L	5 mg/L	60	98.6	–	Xu et al. (2013)
H_3_PW_12_O_40_/ Activated clay	Methyl orange	a 40 W UV light tube (*λ*_max_ = 365 nm)	500 mL	2	1.5 g/L	10 mg/L	60	78.9	0.7 mol/L H_2_O_2_	Wei et al. (2012)
H_3_PW_12_O_40_/La-TiO_2_	Imidacloprid	A 300 W Xe lamp (*λ*_max_ ≥ 365 nm)	50 mL	–	30 mg	10 mg/L	60	98.17	–	Changgen, Gang & Xia (2013)
H_3_PW_12_O_40_-TiO_2_/Bentonite	Methyl orange	Two 15 W UV lamps (*λ*_max_ = 253.7nm)	–	initial pH of methyl orange solution	1,000 mg/L	10 mg/L	120	82.7	–	Zhang et al. (2015)
H_3_PW_12_O_40_/TiO_2_ film	Bisphenol A	A 300W Xe lamp (equipped with IR cut filter, *λ* = 320–780 nm)	100 mL	8.2	–	5 mg/L	240	≈100	–	Lu et al. (2013)
H_3_PW_12_O_40_/TiO_2_	Dinitrotoluene	A 300 W Xe lamp (*λ* = 250–380 nm)	50 mL	2	0.8 g/L	40 mg/L	240	95	–	Feng, Shang & Liu (2014)
H_3_PW_12_O_40_/SiO_2_	Rhodamin B	A 500 W Xe lamp	–	2.5	0.8 g	10 mg/L	120	97.7	–	Yang et al. (2012)
H_3_PW_12_O_40_/TiO_2_	Nitrobenzene	A 500 W tungsten light (*λ* = 400–760 nm)	25 mL	–	10 mg	20 mg/L	390	94.1	–	Wang, Huang & Yang (2010)
H_3_PW_12_O_40_/Ag-TiO_2_	Sulfamethoxazole	A 500 W Xe lamp (equipped with an IR and 400 nm cut filter, *λ* = 400–680 nm)	100 mL	6.8	200 mg	40 mg/L	240	97.8	–	(Xu et al., 2012)
Ag/Ag_x_H_3−*x*_PMO_12_O_40_	Methyl orange	A 300 W Xe lamp (equipped with 420 nm cut-off filter, *λ*>420 nm)	20 mL	1	20 mg	20 mg/L	60	100	–	Shi et al. (2016)
H_3_PW_12_O_40_/TiO_2_	Methyl orange	A 300 W Xe lamp (*λ* ≥ 365 nm)	50 mL	2	0.6 g/L	10 mg/L	18	100	–	Feng & Shang (2012)
H_3_PW_12_O_40_/modified cobalt ferrite	Acid Orange 95	A 9 W (UV-C)	800 mL	–	0.01g	10 mg/L	30	91	–	Mahmoodi et al. (2016)
	Acid Red 18							99		
	Direct Red 81							96		
PW_12_O_40_^3−^ immobilized on an anionic exchangeresin	Rhodamine B	A 500 W halogen lamp (equipped with a 450 nm cut-off filter, visible light)	60 mL	2.5	–	0.02 mM	240	>95	In presence of 2 mM H_2_O_2_	Lei et al. (2005)
cucurbit[6]uril- *α*-Keggin type polysilicontungstate anions	Methyl orange	A 500 W Xe lamp (equipped with a 420 nm cut-off filter)	–	2.5	0.5 g/L	10 mg/L	120	95.6	In presence of 1.5 mM H_2_O_2_	Cao et al. (2011)
								93.6	–	
TiO_2_-NH_2_-H_3_PW_12_O_40_-Au	Congo red	A 150 W Xe arc lamp	10 mL	–	12 mg	10 μ M	30	77	–	Pearson et al. (2011b)
Ag@Ag_x_H_3−*x*_PW_12_O_40_	Methylene Blue	A 300 W Xe arc lamp (*λ*>400 nm)	250	–	–	12 mg/L	120	≈100	–	Zhou et al. (2013)
HPW-yttrium-TiO_2_	Methyl Orange	A 300 W Xe lamp (*λ*>365 nm)	50 mL	1	30 mg	10 mg/L	21	96.6	–	Yajun, Kecheng & Changgen (2013)
PCPs/POM host–guest compound ([Cu(II)_2_Cu(I)_3_(OH)_4_(H_2_O)_2_(TPT)_4_][PW_12_O_40_])	Methyl Orange	A 150 W Xe lamp	250 mL	6.3	0.15 g	15 mg/L	150	91	In presence of 1.5 mM/L H_2_O_2_	Fu et al. (2012)
H_3_PW_12_O_40_/In_2_O_3_	Methylene blue	A 400 W high-pressure Hg lamp (*λ* = 200-400 nm)	10 mL	4.3	20 mg	10 mg/L	15	80	Oxygen flow rate of 5 mL/ min	Salavati & Saedi (2014)
	Solophenyl red-3BL					40 mg/L		49		
	Nylosan black 2-BL-acid					80 mg/L		56		
	Methyl orange					20 mg/L		26		
	Bromothymol blue					40 mg/L		44		
PW_11_O_39_Mn^II^(H_2_O)^5−^/ D301R resin	Rhodamine B	A 200 W metal halide lamp (equipped with a 420 nm cut-off filter)	250 mL	–	100 mg	10 μ M/L	40	100	–	Hua et al. (2014)
SiW_11_/TiO_2_	Rhodamine B	A 125 W high-pressure Hg lamp (*λ*_max_ = 313.2 nm)	80 mL	–	0.015 mM	0.1 mM	180	>90	–	Yang et al. (2003a)
GeW_11_/TiO_2_	Rhodamine B						180	>90		
PW_11_/TiO_2_	Rhodamine B						80	94.4		
	Methyl orange						180	>80		
	Erythrosine B. S.						180	90		
H_3_PW_12_O_40_/TiO_2_	Acid brilliant red 3R	–	–	–	–	–	–	91	–	Sun et al. (2006)
H_3_PW_12_O_40_/TiO_2_/SiO_2_	Rhodamine B	–	–	1	0.2 g	–		>95	–	Wang & Niu (2007)
H_3_PW_12_O_40_/TiO_2_	Methylene blue	Solar light	200 mL	–	0.4 g	50 mg/L	90	95	–	Lee, Dong & Dong (2010)
H_3_PW_12_O_40_/TiO_2_	Rhodamine B	A 350 W Xe lamp	100 mL	–	100 mg	25 mg/L	240	≈80	–	Piao et al. (2013)
TiO_2_/ZnO/H_3_PMo_12_O_40_	Aniline	42 LED lamps (3.2 V, *λ* = 390 nm)	100 mL	unadjusted	0.05 g	50 mg/L	180	38	In presence of 5 mM/L H_2_O_2_	Taghavi et al. (2018b)
TiO_2_/H_3_PMo_12_O_40_								26		
ZnO/H_3_PMo_12_O_40_								45		
TiO_2_/ZnO/H_3_PMo_12_O_40_	Aniline	Two 11 W low-pressure Hg lamps (*λ*_max_ = 254 nm)	100 mL	unadjusted	0.05 g	50 mg/L	180	71	–	Taghavi et al. (2018c)
								74	In presence of 5 mM/L H_2_O_2_	
TiO_2_/H_3_PMo_12_O_40_								72	–	
								77	In presence of 5 mM/L H_2_O_2_	
ZnO/H_3_PMo_12_O_40_								75	–	
								79	In presence of 5 mM/L H_2_O_2_	

In some studies, the optical properties of the composite including a photocatalyst in combination with HPA were better than the case of using a photocatalyst and HPA alone (Changgen, Gang & Xia, 2013; Shi et al., 2012; Yang et al., 2004; Zhang et al., 2015). In most cases, the composite had a stronger photon absorption capability compared with its ingredients (Feng, Shang & Liu, 2014; Lu et al., 2012; Zhang et al., 2015). A red shift was observed in the UV-Vis diffuser reflectance spectra (DRS) for various composites, due to the effect of the Keggin unit and other doping materials on the electronic properties of the photocatalyst (Changgen, Gang & Xia, 2013; Lu et al., 2013; Salavati, Tavakkoli & Hosseinpoor, 2012; Shi et al., 2012; Yang et al., 2004). Doping the photocatalyst by HPA has resulted in an increase in the absorption in the visible region along with a high light harvesting efficiency by expanding the light response region of composite (Lu et al., 2013).

HPA can influence the electron transformation and act as the electron shuttle in light illuminating photocatalyst (Shi et al., 2012). Thus, HPA doping compared with supporting materials can lead to the outstanding photocatalytic activity of a composite photocatalyst. The results of the photocatalytic activity of Keggin-type heteropoly acids on supporting materials in the degradation of organic pollutants in aqueous solutions have been presented in Table 1.

### Mechanism of degradation

Electron–hole pairs are produced and separated when they receive the energy needed for overcoming their mutual electrostatic attraction. Therefore, electrons move to the surface of the photocatalyst where the hole is transferred to the adsorbed hydroxide to create ^∘^OH, and accordingly the electron reacts with O_2_ to form superoxide anion radical (^∘^O_2_^−^) (Ghaneian et al., 2014b; Mahmoodi et al., 2016; Norzaee et al., 2017; Shi et al., 2012).

**Figure 1:**
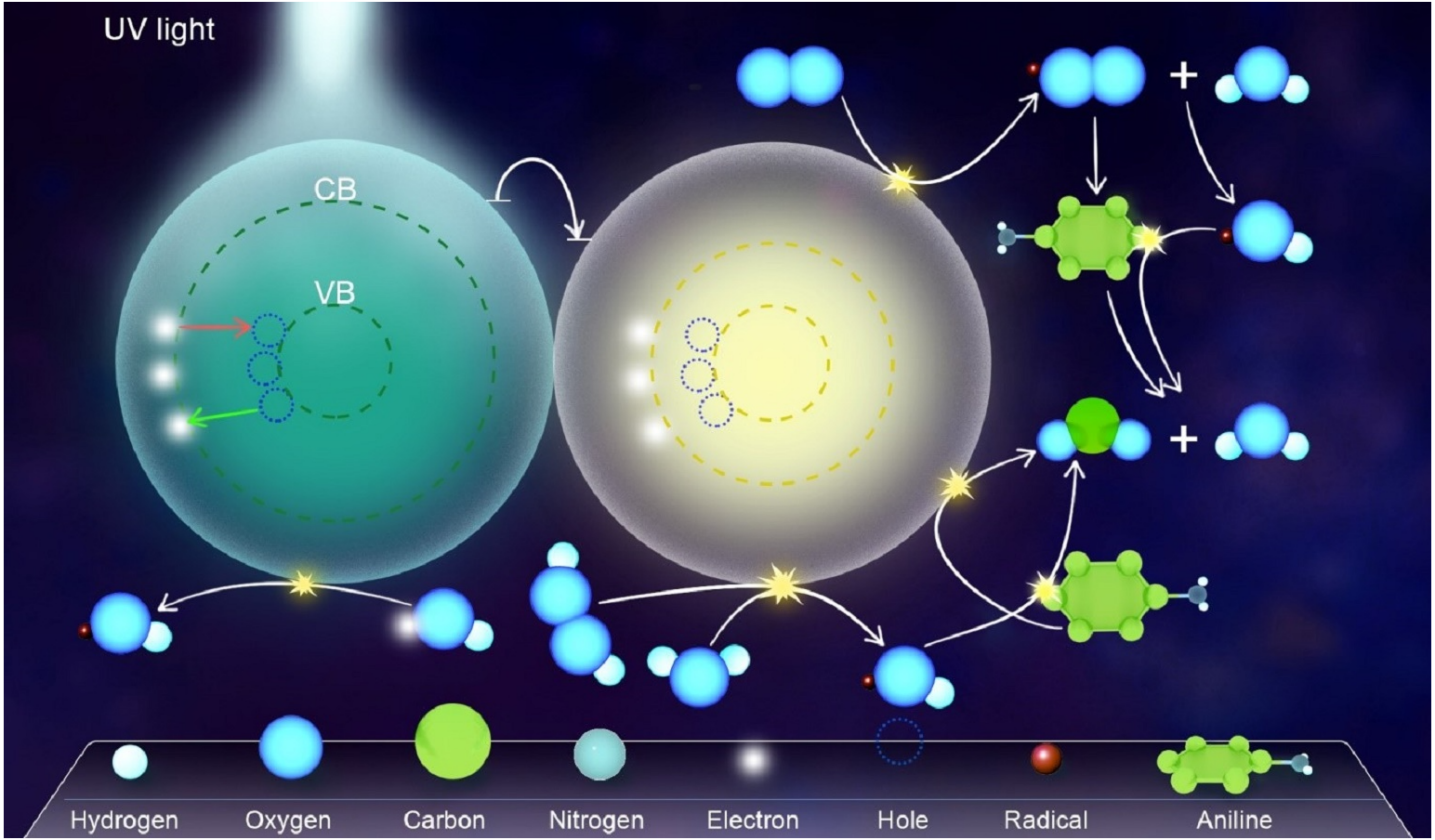
The degradation pathways of aniline as an organic pollutant model, using the composite nanophotocatlyst. Some of the reactions for the degradation of aniline as an organic pollutant model, using the composite synthesized nanophotocatlyst.

The use of HPA as a doping agent creates a new electronic state in the middle of the photocatalyst band gap, leading to a change in the band gap energy (Shi et al., 2012). Thus, it has some advantages such as a decrease in the chance of recombining the electrons and holes. The electrons excited from the valence band of composite because of absorbing UV/visible photon are transferred to the surface of HPA in the composite. In other words, HPA acts as an electron scavenger that prevents the fast recombination of electron–hole pairs, resulting in an increase in the degradation efficiency of the system (Shi et al., 2012). In other words, the photogenerated electron (e^−^) at the semiconductor (SEMI) is trapped into an unoccupied W_5d_ state of the Keggin unit by HPA, which leads to a reduction in heteropoly blue (HPA^−^) (Eqs. (1) and (2)). Given its reducing ability, the HPA^−^ can sensitize the photochemical reduction of O_2_ to produce supperoxides (^∘^O_2_^−^) (Eq. (3)). Specifically, HPA accelerates electron transfer from the semiconductor to O_2_ and retards the recombination electron–hole pairs on the semiconductor. Hence, the enhanced quantum efficiency is achieved in composite photocatalyst, compared to the pure semiconductor. Further, the generated ^∘^O_2_^−^ can interact with the adsorbed water to produce hydroxyl radical (^∘^OH) and oxidize the organic target pollutant Eqs. (4) and (5) (Tabatabaee & Abolfazl Mirrahimi, 2011; Zhang et al., 2013). Furthermore, the photogenerated holes on the semiconductor react with the adsorbed water and hydroxyl ions to yield more ^∘^OH or oxidize the organic pollutant Eqs. (6)–(8) (Ghaneian et al., 2014a; Zhang et al., 2013). As a result, the ^∘^OH as an active species can degrade the organic pollutant Eq. (9) (Tabatabaee & Abolfazl Mirrahimi, 2011; Zhang et al., 2013). Some of these reactions for the degradation of aniline as an organic pollutant model, using the synthesized nanophotocatlyst, are presented in Fig. 1.

(1) \begin{eqnarray*}& & \text{SEMI}\rightarrow ^{\mathrm{h}\nu }\text{SEMI} \left( {\mathrm{h}}^{+}+{\mathrm{e}}^{-} \right) \end{eqnarray*}

(2) \begin{eqnarray*}& & \text{SEMI} \left( {\mathrm{e}}^{-} \right) +\mathrm{HPA}\rightarrow \text{SEMI}+{\mathrm{HPA}}^{-}\end{eqnarray*}

(3) \begin{eqnarray*}& & {\mathrm{HPA}}^{-}+{\mathrm{O}}_{2}\rightarrow \mathrm{HPA}+{}^{\circ }{\mathrm{O}}_{2}^{-}\end{eqnarray*}

(4) \begin{eqnarray*}& {& }^{\circ }{\mathrm{O}}_{2}^{-}+{\mathrm{H}}_{2}\mathrm{O}{\rightarrow }^{\circ }\mathrm{OH}+{\mathrm{OH}}^{-}\end{eqnarray*}

(5) \begin{eqnarray*}& {& }^{\circ }{\mathrm{O}}_{2}^{-}+\mathrm{Org}\rightarrow {\mathrm{Org}}_{\mathrm{ ox}}\rightarrow \rightarrow \text{degradation products}\end{eqnarray*}

(6) \begin{eqnarray*}& & \text{SEMI} \left( {\mathrm{h}}^{+} \right) +{\mathrm{H}}_{2}\mathrm{O}\rightarrow \text{SEMI}+{}^{\circ }\mathrm{OH}+{\mathrm{H}}^{+}\end{eqnarray*}

(7) \begin{eqnarray*}& & \text{SEMI} \left( {\mathrm{h}}^{+} \right) +{\mathrm{OH}}^{-}\rightarrow \text{SEMI}+{}^{\circ }\mathrm{OH}\end{eqnarray*}

(8) \begin{eqnarray*}& & \text{SEMI} \left( {\mathrm{h}}^{+} \right) +\mathrm{Org}\rightarrow \text{SEMI}+{\mathrm{Org}}_{\mathrm{ ox}}\rightarrow \rightarrow \text{degradation products}\end{eqnarray*}

(9) \begin{eqnarray*}& {& }^{\circ }\mathrm{OH}+\mathrm{Org}\rightarrow {\mathrm{Org}}_{\mathrm{ ox}}\rightarrow \rightarrow \text{degradation products}\end{eqnarray*}

Furthermore, the direct irradiation of HPA on supporting materials also leads to the creation of some reactions. The irradiation of H_3_PW_12_O_40_ results in charging transfer from O^2−^ to W^+6^ in *W* − *O* − *W* and creating a pair of the hole (O^−^), along with a trapped electron center (W^5+^), as shown in the following equation (Antonaraki et al., 2010; Yang et al., 2004):

(10) \begin{eqnarray*} \left[ {\mathrm{W}}^{6+}-{\mathrm{O}}^{2-}-{\mathrm{W}}^{6+} \right] \rightarrow ^{\mathrm{h}\nu }{ \left[ {\mathrm{W}}^{5+}-{\mathrm{O}}^{-}-{\mathrm{W}}^{6+} \right] }^{\mathrm{\ast }} \mathrm{i}.\mathrm{e}.,{\mathrm{HPA}}^{\mathrm{\ast }}\end{eqnarray*}

The strong oxidation ability of HPA^∗^, charge transfer-excited state, has been confirmed for degrading organic pollutants (Yang et al., 2004). The hole (O^−^) formed in the charge transfer-excited state of HPA has a strong oxidation ability, which is responsible for oxidizing pollutants (Mahmoodi et al., 2016; Yang et al., 2004). The general reactions involved in degrading organic pollutants in the photocatalytic system containing HPA can be shown by the following equations (Antonaraki et al., 2010; Wei et al., 2012; Zhang et al., 2013):

(11) \begin{eqnarray*}& & {\mathrm{HPA}}^{\mathrm{\ast }}+{\mathrm{H}}_{2}\mathrm{O}\rightarrow {\mathrm{HPA}}^{-}+{}^{\circ }\mathrm{OH}+{\mathrm{H}}^{+}\end{eqnarray*}

(12) \begin{eqnarray*}& {& }^{\circ }\mathrm{OH}+\mathrm{Org}\rightarrow {\mathrm{Org}}_{\mathrm{ ox}}\rightarrow \rightarrow \text{degradation products}\end{eqnarray*}

(13) \begin{eqnarray*}& & {\mathrm{HPA}}^{\mathrm{\ast }}+\mathrm{Org}\rightarrow {\mathrm{HPA}}^{-}+{\mathrm{Org}}_{\mathrm{ ox}}\rightarrow \rightarrow \text{degradation products}\end{eqnarray*}

(14) \begin{eqnarray*}& & {\mathrm{HPA}}^{-}+{\mathrm{O}}_{2}\rightarrow \mathrm{HPA}+{}^{\circ }{\mathrm{O}}_{2}^{-}\end{eqnarray*}

(15) \begin{eqnarray*}& {& }^{\circ }{\mathrm{O}}_{2}^{-}+\mathrm{Org}\rightarrow {\mathrm{Org}}_{\mathrm{ ox}}\rightarrow \rightarrow \text{degradation products}\end{eqnarray*}

In the presence of hydrogen peroxide, the HPA^∗^ can produce hydroxyl radicals in reaction with H_2_O_2_, which is transferred to the reduced state based on the equations as follows (Wei et al., 2012):

(16) \begin{eqnarray*}& & \mathrm{HPA}\rightarrow ^{\mathrm{h}\nu }{\mathrm{HPA}}^{\mathrm{\ast }}\end{eqnarray*}

(17) \begin{eqnarray*}& & 2{\mathrm{HPA}}^{\mathrm{\ast }}+{\mathrm{H}}_{2}{\mathrm{O}}_{2}\rightarrow 2{\mathrm{HPA}}^{-}+{2}^{\circ }\mathrm{OH}\end{eqnarray*}

Ultraviolet irradiation of H_2_O_2_ is another pathway for generating hydroxyl radical in the presence of H_2_O_2_.

(18) \begin{eqnarray*}{\mathrm{H}}_{2}{\mathrm{O}}_{2}\rightarrow ^{\mathrm{h}\nu }{2}^{\circ }\mathrm{OH}\end{eqnarray*}

Some studies emphasized the role of reactive species and its contribution to each in the presence of scavengers, (Lu et al., 2013; Norzaee et al., 2018). For example, Mahmoodi et al. (2016) evaluated the effect of some anions such as NaCl, NaHCO_3_, and Na_2_SO_−4_ on the photocatalytic decolorization of Acid Orange 95, Acid Red 18, and Direct Red 81 by using H_3_PW_12_O_40_/modified cobalt ferrite composite and observed the retarding effect of anions taken place through their reaction with ^∘^OH as well as hole based on the Eqs. (19)–(22). They interpret the dominant effect of holes and ^∘^OH radicals by the fact that owing to the synergistic effect of POM and cobalt ferrite, photoinduced electrons in cobalt ferrite are excited and captured by POM. As a result, a decrease occurs in the production of superoxide anion radical produced via the interaction between photoinduced electrons and oxygen molecules adsorbed on the surface of composite (Mahmoodi et al., 2016).

(19) \begin{eqnarray*}& & \mathrm{OH}+{\mathrm{Cl}}^{-}{\rightarrow }^{\circ }\mathrm{Cl}+{\mathrm{OH}}^{-}\end{eqnarray*}

(20) \begin{eqnarray*}& {& }^{\circ }\mathrm{OH}+{\mathrm{HCO}}_{3}^{-}{\rightarrow }^{\circ }{\mathrm{CO}}_{3}^{-}+{\mathrm{H}}_{2}\mathrm{O}\end{eqnarray*}

(21) \begin{eqnarray*}& {& }^{\circ }\mathrm{OH}+{\mathrm{SO}}_{4}^{2-}{\rightarrow }^{\circ }{\mathrm{SO}}_{4}^{-}+{\mathrm{OH}}^{-}\end{eqnarray*}

(22) \begin{eqnarray*}& & {\mathrm{Cl}}^{-}+{\mathrm{h}}^{+}\rightarrow {\mathrm{Cl}}^{\circ }\end{eqnarray*}

Furthermore, an order of SO_4_^2−^>Cl^−^>NO_3_^−^ has been reported for the influential extent of the anion in reducing degradation Rhodamine B (Hua et al., 2014). The hydroxyl radical also has been represented as the main reactive species in photocatalytic degradation of bisphenol A by H_3_PW_12_O_40_/TiO_2_ film (Lu et al., 2013).

On the contrary, Shi et al. (2016) rejected the significant role of ^∘^OH species on photocatalytic degradation of methyl orange by Ag/Ag_x_H_3−*x*_PMO_12_O_40_ under visible light irradiation and introduced ^∘^O_2_^−^ and h^+^ radicals as the main reactive species in the process. Niu & Hao (2011) also considered the most contribution in degradation Methyl orange for holes.

In some studies, the heteropoly acid leachate from composites also investigated. The use of H_3_PW_12_O_40_/In_2_O_3_ composite photocatalyst in the photocatalytic degradation of methylene blue during four cycles resulted in a leaching of 0.2–1.4% W. Accordingly, a strong coordination communication was observed between the HPA and the In_2_O_3_ facade. The leaching of W was decreased with the increase of application cycle (Salavati & Saedi, 2014). A 2% leaching or loss of POM was reported for H_3_PMo_12_O_40_/MnO_2_ composite photocatalyst after using the fifth cycle (Kannan et al., 2011). Qu, Guo & Hu (2007) found W concentration within the range of 1.1–3.3 mg/L in the final treated solution by H_3_PW_12_O_40_/ZrO_2_, which was negligible with respect to its initial concentration. Finally, a strong interaction was reported between the Keggin unit and ZrO_2_ support with the unchangeable photocatalytic activity of composite for the three-time cycle.

### Effect of initial pH of the solution

The pH of the solution plays an important role in the photocatalytic degradation process (Taghi Ghaneian et al., 2016). Most studies in this regard report a good photocatalytic activity of composite photocatalysts including HPA toward organic pollutant in an acidic medium in a pH range of 1–2.5 (Table 1). Hence, we can infer the partial change of the HPW structure from PW_12_ into PW_11_ at high pH values (Qiu, Zheng & Haralampides, 2007). The surface of the composite including PW_12_ in the outermost layer carries a negative charge due to PW_12_, which results in accelerating the transfer rate of holes and facilitating the separation of electron–hole pairs. As a result, the photogenerated electrons could have enough time to react with the adsorbed O_2_ on the composite surface to yield reactive oxygen species (^∘^O_2_ or H_2_O_2_). As previously mentioned, holes can react with the adsorbed water to generate ^∘^OH. Therefore, decomposition of HPW is responsible for the relatively low photocatalytic activity at neutral and alkaline media (Niu & Hao, 2011).

However, there are some reports with conflicting results in published works, with some of them discussed in detail below.

Hua et al. (2014) observed that the degradation efficiency of rhodamine B increased with an increase in the initial pH ranging 2.5–7 in the homogeneous system (PW_11_Mn), but the vice versa happened in the heterogeneous system (PW_11_Mn/D301R resin). Considering a red shift in the maximum visible absorption peak of rhodamine B in presence of PW_11_Mn, this result is related to the interaction between the catalyst and rhodamine B in the homogeneous system.

Moreover, Changgen, Gang & Xia (2013) reported that the initial pH of the solution within a range of 1–5.88 plays no significant role in degrading imidacloprid by H_3_PW_12_O_40_/La-TiO_2_ nanocomposite. Further, Wei et al. (2012) confirmed the negligible effect of pH on degradation of methyl orange using activated clay-supported HPW. This effect was observed as a decrease in degradation efficiency with an increase of pH in a range of 1.0–7.0.

Through using H_3_PW_12_O_40_/Ag-TiO_2_, an increase took place in the degradation of sulfamethoxazole after increasing the pH up to neutral to alkaline conditions (pH = 6.8–8.7), probably due to the changes in sulfamethoxazole acid–base species and an increase in its adsorption on composite photocatalyst (Xu et al., 2012).

HPW composites in combination with H_2_O_2_ is found in a wide range of initial pH of the solution in the photocatalytic degradation of organic pollutants (Wei et al., 2012).

The maximum degradation of bisphenol A by H_3_PW_12_O_40_/TiO_2_ composite catalyst was found at pH 8.2. The increase in photocatalytic activity of the composite catalyst at an alkaline condition (pH = 8.2) compared with acid solution is attributed to an increase in the number of OH reacting with photoinduced holes on the surface of the composite and a consequent increase in hydroxyl radicals generated for the degradation of bisphenol A. On the other hand, the decrease in efficiency at higher pHs (pH = 10.2) is attributed to ionization of bisphenol A and generation of bisphenolate anion (p*K*a = 9.6–10.2), which leads to the electrostatic repulsion between negatively surface charged composite (pH_pzc_ of TiO_2_ = 6.25) and bisphenolate anion (Lu et al., 2013). Elsewhere, this composite photocatalyst was used in degrading rhodamine B in which the best results were observed in acidic solutions (pH = 4.4) (Lu et al., 2012).

### Effect of photocatalyst dosage

Photocatalyst dosage is regarded as another factor playing a significant role in the photocatalytic process. This dosage should be considered in optimizing the operational conditions. Some studies indicated that an increase in photocatalyst dosage could result in increasing the photocatalytic efficiency (Yang et al., 2012). Therefore, an increase in the photocatalyst dosage does not always lead to the enhancement of photocatalytic activity (Hua et al., 2014; Wang, Huang & Yang, 2010; Yang et al., 2012). An excessive dosage increase, however, may decrease the photocatalytic efficiency. It seems that surplus photocatalyst results in scattering the photons in the solution and decreasing the photons that reach the surface of photocatalyst (Feng & Shang, 2012; Xu et al., 2012; Yang et al., 2012). Some other researchers reported the same results for various composites (Feng & Shang, 2012; Wei et al., 2012; Xu et al., 2012). In contrast, (Changgen, Gang & Xia, 2013) did not report a decrease in photocatalytic activity H_3_PW_12_O_40_/La-TiO_2_ composite with an increase of photocatalyst dosage within the range of 200–800 mg/L. An explanation for this result is that the maximum applied dosage is still in the optimum range of photocatalyst dosage based on other reports (Wei et al., 2012; Xu et al., 2012).

### Effect of pollutant concentration

Several studies have shown that an increase in pollutant concentration leads to a decrease in photocatalytic efficiency through different ways (Feng & Shang, 2012; Lu et al., 2013; Mahmoodi et al., 2016; Niu & Hao, 2011; Norzaee et al., 2017; Xu et al., 2012; Yang et al., 2012).

More pollutant and intermediate products molecules are accumulated on the surface of photocatalyst when an excessive increase takes place in the initial concentration. Accordingly, the generation of reactive oxygen species is reduced because of inhibiting the adsorption of the incident photon for active sites (Lu et al., 2013; Niu & Hao, 2011). As for the dyes, the higher concentration of dye makes the color of solution very deeper and thus results in limiting the penetration of light to the solution depth and reaching the surface of photocatalyst (Feng & Shang, 2012). Besides, a constant intensity of light source and illumination time leads to a constant amount of the generated radicals (Norzaee et al., 2017). Hence, surplus dye molecules cannot be degraded and are represented as a low degradation efficiency (Niu & Hao, 2011).

Nevertheless, the low concentration of target pollutant may lead to a weak interaction between pollutant and composite photocatalyst. Wang, Huang & Yang (2010) reported this point as a reason for low efficiency in degradation of nitrobenzene by H_3_PW_12_O_40_/TiO_2_ composite photocatalyst in initial concentration 10 mg/L and increasing the efficiency through enhancing the initial concentration of nitrobenzene.

### Effect of oxidants

In some studies, oxidants such as H_2_O_2_ have been applied to improve photocatalytic activity. Increasing the H_2_O_2_ concentration from 0.2 to 0.7 mol/L in a UV/ H_3_PW_12_O_40_/activated clay system could raise the degradation of methyl orange. However, a larger increase in the oxidant dosage results in decreasing the degradation efficiency of methyl orange (Wei et al., 2012).

Cao et al. (2011) reported that the photocatalytic degradation process is accelerated in the presence of H_2_O_2_. In addition, it has been evidenced that addition of H_2_O_2_ to the photocatalytic system leads to an increase in the hydroxyl radicals produced in the system, which is regarded as a reason for enhancing the degradation rate (Wei et al., 2012).

H_2_O_2_ at high concentrations has a negative effect on photocatalytic degradation rate by decreasing the number of hydroxyl radicals in solution because of acting as a hydroxyl radical scavenger at a higher level of concentration. A decrease in hydroxyl radical due to the high concentration of H_2_O_2_takes place through the following reactions (Wei et  al.,  2012):

(23) \begin{eqnarray*}& & {\mathrm{H}}_{2}{\mathrm{O}}_{2}+{}^{\circ }\mathrm{OH}\rightarrow {\mathrm{H}}_{2}\mathrm{O}+{}^{\circ }\mathrm{H}{\mathrm{O}}_{2}\end{eqnarray*}

(24) \begin{eqnarray*}& & \mathrm{H}{\mathrm{O}}_{2}+{}^{\circ }\mathrm{OH}\rightarrow {\mathrm{H}}_{2}\mathrm{O}+{\mathrm{O}}_{2}\end{eqnarray*}

### Effect of heteropoly acid loading

Various concentrations of heteropoly acid can change the photocatalytic activity of composites (Changgen, Gang & Xia, 2013; Lu et al., 2013). The HPW loading on La-TiO _2_nanoparticle (0.3%) within the range of 10–20% resulted in increasing the photocatalytic activity of nanocomposite while the further increase in HPW loading led to a slight decrease (Changgen, Gang & Xia, 2013). Zhang et al. (2015) found same results in preparing H_3_PW_12_O_40_-TiO_2_/Bentonite and the highest photocatalytic activity achieved at a molar ratio of 0.5:100 for HPW and TiO_2_, respectively. In addition, the rate constant *k* for an HPW loading of 20% in H_3_PW_12_O_40_-TiO_2_ composite was 1.4 times that for an HPW loading of 30%, and approximately 3 times that of an HPW loading of 10% and 40% (Feng & Shang, 2012).

Other studies reported similar results for various composite photocatalysts (Lu et al., 2013; Zhang et al., 2013). The first increase in photocatalytic activity along with an increase in a doping of HPA is attributed to the increase in captured electrons by HPA, as an electron scavenger, a consequent delay in recombination electron–hole pairs, and an increase in the photocatalytic activity (Lee, Dong & Dong, 2010; Zhang et al., 2015). However, a high increase in the doping intensity of HPA is not always favored. A high amount of HPA causes to increase the available electron traps provided by HPA and the distance between electron traps will decrease. Therefore, an excessive amount of HPA in the composite can provide a place for recombining the photogenerated electron–hole pairs, leading to a decrease in photocatalytic activity of composite photocatalyst (Zhang et al., 2013; Zhang et al., 2015). Another reason for this phenomenon might be a reduction in specific areas of composite photocatalyst and accordingly less active sites for light and pollutant introduction (Feng & Shang, 2012; Lu et al., 2013; Zhang et al., 2013).

### Photocatalyst recovery

The H_3_PW_12_O_40_/TiO_2_ composite synthesized by combining sol–gel technology using a nonionic surfactant P123 as a structure directing agent with solvothermal treatment demonstrated a constant photocatalytic activity after three sequent cycles. Accordingly, the prepared composite photocatalyst using this method has a high stability (Feng, Shang & Liu, 2014). Shi et al. (2016) reported the same result for Ag/Ag_x_H_3−*x*_PMO_12_O_40_ after degrading methyl orange for four cycles. Further, Xu et al. (2012) reported the use of H_3_PW_12_O_40_/Ag-TiO_2_ composite in degrading Sulfamethoxazole with an insignificant decrease in photocatalytic activity after three cycles. The degradation rate for the first, second, and third cycles were 97.6, 91.7, and 87.7%, respectively. Similar results were reported for H_3_PW_12_O_40_/modified cobalt ferrite composite in the degradation of some dyes (Mahmoodi et al., 2016). This result is consistent with those reported in (Feng, Li & Liu, 2012; Zhang et al., 2013). However, Niu & Hao (2011) obtained different results for five cycles by reusing H_3_PW_12_O_40_/TiO_2_ in the decomposition of methyl orange. They reported that an increase could take place in decomposition efficiency after an increase in reusing cycles, due to the change in photocatalyst surface property and the bound water produced on the recycled photocatalyst.

### Outlook on challenge and perspective

From the catalytic activity point of view, the previous sections showed that application of heteropoly acids on supporting materials in the photocatalytic process for the removal of organic pollutants has been an effective move in this field of study. Increasing photocatalytic activity for composite materials and overcoming problems in regard to the separation of heteropoly acids from the final solution are the most significant achievements of the application of heteropoly acids on supporting materials. Although some researchers also used SiO_2_, BiVO_4_, Au, Mg_3_Al-LDH, ZrO_2_, MnO_2_ as supporting materials, most works to date have focused on TiO_2_ as supporting materials. Future works should focus on materials that have not yet been used such as Fe_2_O_3_ to develop composites with desirable separation properties as well as the biotoxicity of treated wastewater, the changes in the environmental toxicity of synthesized composites, and applying facilitating methods for the composite photocatalysts such as HPA in a wider range of pH.

## Conclusion

An overview was conducted on the use of Keggin-type HPA on solid supports such as nanophotocatalysts, resins, rare earth elements (REEs), clays, and other materials in the photocatalytic degradation of water and wastewater pollutants. The findings revealed a relatively high photocatalytic activity of composite photocatalysts toward organic pollutants. In addition, the combination of HPA with other semiconductors had some advantages, such as a delay in the recombination of electron-hole pairs, enhanced absorption of UV light for the composite, increased light harvesting of the composite in the visible region, and accelerated electron transfer of semiconductor, especially TiO_2_ toward O_2_. For other materials, some advantages such as recycling HPA and preventing secondary pollution because of the solubility of HPA can be mentioned. Moreover, some aspects of the synthesis and application of supported HPA were reviewed in the present study. However, further research is needed for the use of easily recyclable supporting materials, the biotoxicity of treated wastewater, the changes in the environmental toxicity of synthesized composites, and applying facilitating methods for the composite photocatalysts such as HPA in a wider range of pH.

## Additional Information and Declarations

### Data Availability

The following information was supplied regarding data availability: This article is a literature review and did not generate any data.
